# Registered nurses´ views on telephone nursing for patients with respiratory tract infections in primary healthcare – a qualitative interview study

**DOI:** 10.1186/s12912-020-00459-1

**Published:** 2020-07-14

**Authors:** Elenor Kaminsky, Ingrid Edvardsson Aurin, Katarina Hedin, Lisbet Andersson, Malin André

**Affiliations:** 1 Department of Public Health and Caring Sciences, Uppsala University, Uppsala, Sweden; 2 Department of Research and Development, Region Kronoberg, Växjö, Sweden; 3 Department of Clinical Sciences in Malmö, Family Medicine, Lund University, Malmö, Sweden; 4 Futurum, Region Jönköping County, and Department of Health, Medicine and Caring Sciences, Linköping University, Linköping, Sweden; 5 Department of Health and Caring Sciences, Faculty of Health and Life Sciences Linnaeus University, Linnaeus, Sweden

**Keywords:** Registered nurse, Telephone nursing, Telephone assessments, Respiratory tract infections, Primary healthcare, Qualitative study

## Abstract

**Background:**

Telephone nursing in primary healthcare has been suggested as a solution to the increased demand for easy access to healthcare, increased number of patients with complex problems, and lack of general practitioners. Registered nurses’ assessments may also be of great importance for antibiotic prescriptions according to guidelines. The aim of this study was to describe registered nurses’ views of telephone nursing work with callers contacting primary healthcare centres regarding respiratory tract infections.

**Methods:**

A descriptive, qualitative study was performed through interviews with twelve registered nurses in Swedish primary healthcare.

**Results:**

The overarching themes for registered nurses’ views on telephone nursing were captured in two themes: professional challenges and professional support. These included three and two categories respectively: Communicate for optimal patient information; Differentiate harmless from severe problems; Cope with caller expectations; Use working tools; and Use team collaboration. Optimal communication for sufficiently grasping caller symptoms and assess whether harmful or not, without visual input, was underlined. This generated fear of missing something serious. Professional support used in work, were for example guidelines and decision support tool. Colleagues and teamwork collaboration were requested, but not always offered, support for the interviewed registered nurses.

**Conclusions:**

The study deepens the understanding of telephone nursing as an important factor for decreasing respiratory tract infection consultations with general practitioners, thus contributing to decreased antibiotic usage in Sweden. To cope with the challenges of telephone nursing in primary healthcare centres, it seems important to systematically introduce the use of the available decision support tool, and set aside time for inter- and intraprofessional discussions and feedback. The collegial support and team collaboration asked for is likely to get synergy effects such as better work environment and job satisfaction for both registered nurses and general practitioners. Future studies are needed to explore telephone nursing in primary healthcare centres in a broader sense to better understand the function and the effects in the complexity of primary healthcare.

## Background

Primary healthcare in the Western world faces vast challenges [1], with increasing demands for easy access. In addition, the number of patients with chronic and complex problems have increased, as have the difficulties in recruiting general practitioners (GPs). A suggested solution to these problems is the introduction of telephone nursing assessments by registered nurses (RNs) to reduce the numbers of face-to-face contacts and out-of-hours visits by GPs [2].

Sweden has a long tradition of telephone nursing in primary healthcare. When primary healthcare in the 1970s expanded, there was a scarcity of Swedish GPs. Thus, RN telephone nursing has become a central part of the primary healthcare system. This entails that RNs, based on caller symptoms, assess whether callers contacting primary healthcare centres (PHCCs) need to see a GP, be given self-care advice and stay home, or be recommended to visit other health services. The RN telephone work is commonly performed as part of the working day, in addition to seeing patients face-to-face. Most RNs working in Swedish primary healthcare have a specialist education, e.g. in district nursing, with their own PHCC surgery. Besides this, they often also have special assignments, e.g. child healthcare, diabetic, asthma or health-promoting appointments.

Today, patients are encouraged to call RNs at PHCCs as the first step for healthcare contacts. ‘Drop in’ appointments at PHCCs are also often available, where RNs make a first decision as to whether callers need a GP appointment or not. Swedish healthcare is publicly financed, and telephone calls to RNs are free of charge. GPs and RNs are salaried and typically work in group practices. In addition to PHCC telephone nursing during office hours, a national service, Swedish Healthcare Direct, offers telephone nursing around the clock [3].

Infections are common causes for primary healthcare contacts across Europe, accounting for around 30% of all visits to a GP in Sweden, whereof respiratory tract infections (RTI) constitute around 60%. In just under half of these GP visits, RTI patients are prescribed antibiotics [4]. Problems concerning RTI constitute a major portion of the calls for telephone assessments out-of-hours [5]. National guidelines for management of common infections have been in place in Sweden for 20 years, as well as a national web-based decision support tool (DST) available to aid RN assessments of callers’ health problem urgency [6]. The DST was originally developed for telephone nursing at Swedish Healthcare Direct and is nationally updated on a regular basis in line with current guidelines.

Results from an earlier study suggest that RN’s telephone assessments at PHCCs during office hours may be of great importance for the prescription of antibiotics according to guidelines [7]. Most RTIs are self-healing, where proper advice for self-care is the best option [8, 9]. At the same time, callers who would benefit from antibiotic treatment, and those with signs of a serious disease, must be identified for appropriate action. Thus, well-functioning RN telephone assessments of callers with RTI is important. Earlier studies suggest that DST, guidelines, local routines, continuous medical education (CME), as well as well-functioning cooperation with PHCC professionals and feedback, may facilitate correct RN assessments [7, 10].

Most research has examined the use of telephone nursing in an out-of-hours context [3, 11–13]. In recent years, some studies from UK report the effects of introducing telephone nursing in primary healthcare during office hours [14–17] but research concerning the RNs’ experience of telephone work is sparse [18]. Therefore, the aim of this study is to describe RNs’ views of telephone nursing work with callers contacting PHCCs regarding respiratory tract infections.

## Method

### Study design

This is a descriptive and inductive qualitative interview study. Data was collected through semi structured interviews with open-ended questions to stimulate the RN respondents own narratives [19]. The interviews were 30 to 40 min long*,* recorded and transcribed verbatim. An interview guide with open-ended questions was used. It contained questions regarding the telephone work, how caller needs and problems can be met and managed, if they used any available local guidelines and routines, how collaboration with GPs worked, and if CME was offered. Follow-up questions were asked to gain a deeper understanding.

### Study population

A strategic sample of 12 RNs, working at eight PHCCs in southern and mid-Sweden, including daily work with telephone nursing, were interviewed for the study. Their working time with telephone nursing varied between and within PHCCs. The interviews were performed in January and February 2014, as part of a research project studying antibiotic prescription in PHCCs [6]. In that study, however, only six PHCCs were studied. The interview questions included: Can you please tell me about your telephone nursing work with callers contacting your PHCC about respiratory tract infections?; How are the assessments performed in these calls?; and What is difficult and which facilitators do you use in work? Further, were follow-up and probing questions asked, such as: Could you please tell me more about this; What do you mean by …; and Could you please repeat and explain … - to clarify and deepen the informants’ answers.

The twelve RN interviews were randomly selected from every third interview out of the 26 previous project interviews, i.e. the first, fourth, seventh, tenth, thirteenth, sixteenth, ninteenth, twenty-second, twenty-fifth, resulting in nine interviews. In addition, to obtain a strategic sample representing PHCCs in regard to urban/rural areas, and prescription of antibiotic to a high/low extent, another three interviews were added to the first nine interviews. In all, the study data thus involved twelve interviews. See Table 1 for participating RN characteristics.

**Table 1:** Description of characteristics of the interviewed RNs. *n* = 12

Age of the interviewed RNs’ median (range)	48.5 (32–65)
Specialized district nursing (number)	11
Work experience, years in a PHCC median (range)	14.5 (4–25)
Working at a PHCC in urban areas (number)	4
Working at a PHCC with antibiotic prescription above average (number)	5

### Data analysis

Qualitative content analysis with an inductive approach was carried out according to Graneheim and Lundman [19]. Hence, no pre-defined categories were used, but instead categories emerged from data. The analysis was performed in a number of steps. First, interview transcripts were read several times to catch the overall meaning. Meaningful units that corresponded to the study aim were then identified and selected from the text. Next, these meaningful units were condensed to a reduced description, i.e. the manifested content, as well as an interpretation of the underlying meaning, i.e. the latent content. The text units were then condensed and coded and finally put together into different categories. All authors analysed the text, independent of each other. The different steps of the analysis were discussed until consensus was reached. An example of the analysis process is shown in Table 2.

**Table 2:** Example of the analysis process

Meaningful unit	Condensation	Code	Category	Theme
So my job in assessments is to distinguish what is like … who comes here and what should be self-care, and of what is coming here, what could be something that is more serious?	My job in assessments is to distinguish what is self-care, and what could be something more serious	Assess the level of care	Differentiate harmless from severity*.*	Professional challenges

### Ethics

The study followed the ethical regulations and guidelines according to Swedish law [20] and conformed to the ethical principles defined in the World Medical Association Declaration of Helsinki [21]. The regional Ethical Review Board in Lund, Sweden (2013/679) approved the study. All interviewed RNs gave informed consent for participation. These were also informed that participation was voluntary and that they could withdraw from the study at any time, without giving a reason. The data have been handled confidentially, and the results are presented in a way that avoid identifying individuals.

## Results

Two themes and five categories emerged from the data regarding describing RNs’ views of telephone nursing work with callers contacting PHCCs about respiratory tract infections. These are presented in Table 3. RN informant quotes are presented in italics and the interview numbers are presented in brackets.

**Table 3:** Categories and themes for the described RNs’ views of telephone nursing work with callers contacting PHCCs regarding respiratory tract infections

Categories	Themes
Communicate for optimal patient information	Professional challenges
Differentiate harmless from severe problems	
Cope with caller expectations	
Use working tools	Professional support
Use team collaboration	

### Professional challenges

During the category analysis, the theme *challenges* emerged from the three categories*: Communicate for optimal patient information, Differentiate harmless from severe problems, Cope with caller expectations.* These all dealt with what RNs found difficult and challenging in their telephone nursing professional work at PHCCs. The three categories under this theme are further presented below.

#### Communicate for optimal patient information

Communication in calls was raised by the interviewed RNs as a difficult task. The lack of visual information and the need to rely solely on verbal caller information increased RNs’ concern. They were worried about risks of misunderstanding the caller or that the caller would misinterpret given advice. They strived for optimal communication, and to obtain a clear patient picture during calls. For this, RNs asked callers a variety of questions, mainly about symptoms to come further in disease detection.

Many RNs tried to figure out if the caller was known to them and whether the symptom description was adequate. The importance of noticing implicit cues, such as background sounds, e.g. a supporting spouse, was underlined. Some callers were reported to describe their symptoms in detail and perhaps exaggerate, in order to get a GP appointment. Contrary to this, others provided brief information and underestimated serious symptoms:

*Some say well, they are not very sick, and some are very sick, though they may not seem very sick, so it is … I think it is basically difficult. Because you can get tricked by those who say, well, it’s not so bad … yes. But they are in fact very sick.* (Interview 7)

Due to lack of observable signs, the RNs reported that they must rely on what they heard. For example, apart from caller descriptions of breathing problems, they also listened to callers’ breathing and coughs. When RNs felt uncertain, they asked GPs at the PHCC for advice. In some PHCCs, RNs could offer face-to-face RN consultations, when the patient condition was unclear.

*That’s what we do, in dubious cases we can … have the possibility to get them over here in some way and make an assessment … then you are able to see their skin colour and lots of other things.* (Interview 4)

#### Differentiate harmless from severe problems

Another demanding assignment highlighted by the RNs, was to make correct assessments regarding symptom severity and whether the caller should see a physician (urgent or non-urgent) or receive RN self-care advice. The necessity to base assessments on thorough medical histories was emphasized. A need for sensitivity, nursing experience, and medical knowledge was also suggested in order to facilitate interpretation and assessments of the callers’ condition over the telephone. The RNs were well aware of the risk of telephone nursing, regarding making the right decision, e.g. concerning if the person could manage with self-care advice, should get a GP appointment at once or could wait a few days. In this respect, the RNs described as functioning as a gatekeeper:

*So my job in the assessment is to distinguish what is like … who comes here and what should be self-care, and of what is coming here, what could be something that is more serious?* (Interview 3)

The RNs sometimes made probabilistic assessments for diagnosis or considered other serious differential diagnoses. They feared missing serious diseases. Several described how they thought about possible underlying causes besides an infection when they for example were told about a cough. When self-care advice was given, the caller was prompted to call again if the symptoms did not disappear:

*Well, all breathing problems and everything, and you would think … yes, it is pulmonary embolism and … these rapid ones, but it’s a cough, there are usually some breathing problems then … mm. Yes, I am very generous by saying they can get in touch in case of any small worsening.* (Interview 6)

Regarding assessments, RNs expressed that telephone encounters with parents concerning sick children was the most difficult task in the telephone nursing work. They often felt uncertain about paediatrics and were therefore afraid of making mistakes. It was difficult to get a clear picture as to the degree of the child’s illness, through the interview with the parent. Hence, GP appointments for children were given generously, despite a fully booked schedule. It was also reported to be important to comfort and calm parents:

*I think that I am probably a bit uncertain there, when it comes to small children, so I may more often give them an appointment with the physician, as I feel that I cannot sort it out over the phone when it comes to children.* (Interview 13)

Most of the RNs stated that they valued their work with self-care advice, since they found many respiratory tract infections to be self-healing and wanted to help callers avoid unnecessary antibiotics. The RNs also mentioned callers who were well aware of not using antibiotics unnecessarily, and who did not ask for medication. According to the interviewed RNs, the number of callers with this attitude had increased.

*If you kind of explain what it is … then I think people in general are very good about the fact that you should not take antibiotics unnecessarily, they don’t really nag you*. (Interview 3)

### Cope with caller expectations

Another challenge according to the RNs, was the circumstance whereby some callers were perceived as impatient and required help and cures at once. Even before calling, they had decided to ask for a laboratory test, a GP appointment or antibiotics. Most of the persons experienced as demanding were reported to be young and employed, or on their way to holiday abroad. These people were not receptive to advice according to the RNs.

*I don’t think they are sick enough to be brought in … Yes, I think … if for example it’s someone who does not seem very sick, but … they still want to see a physician, because they want to get well quickly as they will be travelling abroad, etc. And it’s like this … they want penicillin, to get well right away, that’s what they think.* (Interview 1)

The RNs tried to handle the caller’s expectations by discussing and explaining the reasons for symptoms and whether a GP appointment would be necessary or not. Simultaneously, the RNs underlined their ambition to satisfy the callers, comfort them and make them feel listened to. When unable to meet caller expectations through discussion, the RNs sometimes felt forced to book a GP appointment, even though this was against the guidelines.

*Well, the important thing is to make them feel … listened to and they are pleased and they don’t hang up and are dissatisfied or worried or whatever, but you try to satisfy them, that’s what I feel is important.* (Interview 11)

The RNs underlined the importance of understanding and considering cultural aspects. Depending on callers’ cultural backgrounds, some could have other expectations than the ‘normal caller’ regarding healthcare, e.g. other traditions regarding self-care advice or use of antibiotics. In these cases, the RN ambition to give self-care advice was often not well received.

*It’s a cultural issue, and therefore it’s difficult to give self-care advice, as they don’t want to understand that, no, and it’s about seeing a physician, it’s something within them.* (Interview 5)

Parental wishes for a GP paediatric consultation only rarely included a wish for antibiotics according to the interviewed RNs. In these cases, some stated it was equally important to just comfort parents. These had found that many parents were comforted by receiving an RN appointment when their child was ill.

*Yes, at least with parents and their children, I may feel, when they … with the ears then, that they say that you don’t need antibiotics treatment and it’s nice not to have to give their children antibiotics, and the parents think so too, and it’s very trying to do it, and of course you don’t want to give the children medication they don’t need.* (Interview 1)

*Sometimes you think that … bring the children here, a bit because … to calm the parents down.* (Interview 6)

### Professional support

Throughout the category analysis, the theme *support* also emerged from two categories In data. *Use working tools and Use professional collaboration.* Here, RNs talked about how to get professional support in their work via tools such as guidelines and the DST, and to collaborate with professional colleagues. However, not all informants reported to use these job supports. The two categories under the theme support will be further described below.

#### Use working tools

Concerning the RNs’ reported use of work support, their use of and adherence to DST and guidelines seemed to vary. Some used the available DST. Others reported to know about it, but did not use it. Some RNs had not heard of the DST. Regarding guidelines, some read these when needed, while others remembered them by heart:

*I have them (the guidelines) memorised, I ask questions and then … if I need to read up on it, I look it up and leaf through it.* (Interview 3)

When guidelines were explicit, integrated into PHCCs’ local routines, and thus well-known to all PHCC personnel, RNs reported that using them was easier. This made RNs aware of why and with what objectives a certain measure should be taken. When infectious symptom management routines were missing, some RNs reported that they instead tried to figure out what to do in order to fulfil the wishes of individual GPs.

The RNs’ reported that opportunities for support via competence development in CME regarding infectious diseases varied. Some had participated in occasional educational activities, while others desired to be regularly informed. A common opinion was that GPs were offered more opportunities for CME, than RNs. Some RNs reported choosing not to prioritize education on infectious diseases, but rather participated in education in their field of special expertise and responsibility, such as diabetes or smoking cessation:

*I have chosen other fields, yes. I guess it feels like the others have also prioritised other topics. Because it feels more … that* [infectious diseases] *is more for the physicians … but it’s also very important to us, but … the fact that we have chosen topics* [education] *that are for us to do, redressing a wound and … yes, diabetes, smoking cessation, things like that … but of course one would like to do that* [infectious diseases] *as well* (Interview 10)

#### Use team collaboration

Another support in work reported by RNs, was to receive feedback in various ways from other professionals. This was particularly important for dealing with uncertainty regarding an assessment of caller symptoms, after a conversation over the phone and booking a GP consultation. Feedback was considered to be a source for learning for the future. This was managed in several ways by the interviewed RNs. Some read the medical records after GP consultations to find out how the physician had assessed the patient symptoms, as a second opinion to their own judgement. No RN expressed having received feedback from GPs in a systematic way. Instead, this had mostly happened on the RNs’ own initiative, who could ask the GP in question for feedback.

*Sometimes you can go back in the records, if you have a moment to spare, and see what the physician wrote in the medical records … and what happened. I don’t need to speak to the physician directly, as I can read it myself. That’s what you can do. So that’s a development.* (Interview 5)

*We try to tell the physicians that they are welcome to give us feedback about our GP bookings, so we can see if that was right or wrong.* (Interview 5)

When the RNs felt uncertain and wanted advice from a GP about individual callers on the phone, or in their own clinic, they reported they had to ‘disturb’ the GPs in their work. Thus, this was a mutual problem for both RNs and GPs. The RNs stated that they had learned which GPs were OK to contact.

*No, let’s do it while they* [patients] *are still here* [at the RN clinic] *and go see the physician and discuss it, what will we do now, what do you think. Then, we always get a reply.* (Interview 7)

Some PHCCs were reporzted to have regular meetings for RNs, with functioning collegial support, where management of individual caller or patient cases could be discussed. Others lacked support forums for joint discussions with colleagues, concerning assessments and management of diverse infectious disease symptoms.

*Sometimes you feel, maybe, that you would need to meet, maybe just the RNs, and have a discussion, so we are bringing the same message over the phone, you know. A bit more than we are doing at the moment.* (Interview 2)

RNs reporting this, often felt alone in making assessments over the phone. They wished for additional help from colleagues and increased information sharing and recommendations to be used in their work with callers. Hence, there were few opportunities for discussing issues when uncertain. Consequently, unpaid lunch breaks were sometimes used for collegial support, according to the interviewed RNs:

*I guess generally … that we are talking to each other, the RNs. Yes, we do. Yes …* [laughing] *… during our lunch … Now, I have had this and that … in the assessment, and imagine that … this child had been sick for two days, like, and the mother is coming ... we can bring up different issues, what we will do. Yes, we communicate with each other, what did you do then?* (Interview 2)

## Discussion

This qualitative study on RNs’ views of telephone nursing work with callers contacting PHCC regarding respiratory tract infections, resulted in two themes of professional challenges and professional support. The expressed challenges were Communicate for optimal patient information, Differentiate harmless from severe problems, and Cope with caller expectations. The communicated professional support were Use working tools; and Use team collaboration. The most striking finding was that besides highlighting challenges in the telephone nursing work, the interviewed RNs suggested how to get support in their work.

The RNs highlighted the importance of optimal communication in order to make correct assessments. They reported telephone nursing as challenging, regarding the assessment of both who the calling person is and the actual problem, due to relying only on what was heard. Furthermore, these two tasks were interrelated in an intricate way, in line with earlier studies [22]. Thus, training and communication skills seem as important as knowledge of the different guidelines for RTI [23]. The dual roles of telephone nursing have been described as care providers and gatekeepers [24]. Studies from UK primary healthcare of RN assessments during office hours reported a decrease in GP face-to-face contacts, but no decrease in overall clinical contacts [14–17].

In Sweden, telephone nursing has long been obligatory before receiving GP appointments. These are scarce, and consultation times are longer than in most European countries [25]. RNs must thus balance the risks of missing serious cases against taking responsibility for GP workloads [26]. The RNs consequently strived to frequently recommend self-care advice, to avoid unnecessary GP consultations and antibiotic prescriptions. This is in line with reports about telephone RNs’ wish to educate callers and facilitate their learning [27]. The interviewed RNs lowered their threshold for allowing GP appointments concerning children. Second hand consultations, common in paediatric health calls [5], entailed further difficulties regarding assessments [28]. Comforting parents was described as important as curing, well in line with earlier studies [27, 29].

RN telephone nursing has been suggested as crucial for decreasing RTI consultations with GPs, and has thus contributed to the decrease of Swedish antibiotic prescriptions [7, 30]. The number of GP visits for RTI has almost halved for preschool children since late 90s [4, 31]. This change would not have been possible without RNs’ promotion of basic guidelines, which is corroborated by all interviewed RNs confirming the view that many respiratory tract infections are self-healing. Moreover, these should be managed by self-care advice [32], in order to empower patients and reduce healthcare utilisation. This is possible in a healthcare system financed by taxes with well-educated and salaried RNs and GPs.

Surprisingly, the DST and guidelines were only partly known and used, in line with earlier research [22, 33]. In Sweden the national RTI guidelines are implemented through outreach PHCC visits, both for GPs and RNs [30]. However, the guidelines were developed by physicians, focusing on diagnosis and treatment, and thus seldom elaborated on topic relevant for telephone nursing. The modest RN participation in creating the guidelines is astounding in a country with long traditions of PHCC telephone nursing. On the contrary, the national DST is designed for this purpose, and conforms to guidelines. The national DST is mandatory for RN work at Swedish Healthcare Direct but was not systematically introduced in primary healthcare at the time of the study. However, the national DST focuses on caller symptoms, while some RNs, in line with the guidelines, described thinking of diagnoses, e.g. concerning callers with cough. A more consistent use of DST might facilitate shared PHCC routines and teamwork between GPs and RNs, but should probably be complemented with local interprofessional discussions [7]. Strangely enough, no RN mentioned to use patient information on the national healthcare website, 1177 [34], or the Swedish Public Health Agency [35], available in several languages.

Contrary to the RN work at Swedish Healthcare Direct, the interviewed RNs had chosen to work at PHCCs for the possibility of face-to-face work with own patients [36]. The RNs reported that they preferred their main duties, and viewed answering telephone calls as imposed work, which is in line with Murdoch et al. 2015 [18]. Thus, CME for these common problems had low priority, both from an organizational and individual perspective. These circumstances could perhaps be overcome by education and facilitating PHCC adaption and transformation of guidelines among all staff through inter-professional discussions [7, 37]. A previous study [7] revealed that low-prescribing PHCCs regarding antibiotics had reserved time for regular meetings concerning medical issues, and prioritized training within and between professions. Somehow, RNs’ knowledge and competence on telephone nursing issues in PHCCs, where RN self-advice is crucial, is taken for granted by organisations. Thus, beyond continual forums for local CME, proper introductions and preparatory training for recently employed RNs is an urgent measure to establish which is also likely to decrease nursing turnover.

In line with Ahlstedt et al. [38], the interviewed RNs wished for feedback regarding their work. This could be facilitated at PHCCs by scheduling GP and colleague meetings for peer reviews, which no interviewed RN had experienced in a systematic way. Our earlier study as well as several studies emphasize well-functioning inter professional cooperation as the key for knowledge translation and evidence-based practice [7, 39, 40]. In many countries, RNs and GPs work together but the role and function of RN work vary between countries [41]. Close cooperation between RNs and GPs is likely to increase care quality and facilitate feedback [42]. A fruitful collaboration builds on mutual professional respect and trust, based on professional competence [41]. In this respect, the prerequisites are good in Sweden, where health professionals are well-educated. However, this study demonstrates that the RNs had to ask for feedback in order to receive this. The RNs not only wanted this from GPs, but also asked for support possibilities from colleagues and time for discussions within their own profession. These activities could decrease occupational stress and reduce feelings of loneliness in making telephone assessments [43]. At PHCCs with professional collaboration, i.e. open working climate, this had been organised by RN professionals themselves.

Telephone nursing in Swedish primary healthcare has been established for many years, however sparsely researched. The present study is one of a few international studies that explore telephone nursing within PHCCs. The described RNs’ views may thus be valuable, not least for other countries implementing telephone nursing in primary healthcare settings. A strength of the study was that the research team contained both GPs and RNs. Qualitative content analysis was valuable for describing knowledge and understanding of the telephone nursing phenomenon [19]. The interviews were carried out as part of a project with the aim to explore factors important for antibiotics *prescription* [7]. Thus, the focus was not the wider perspective on RN performance or experiences of telephone nursing. No RN researcher was involved in the initial research group. If this had been the case, the interview guide may have been developed differently. Moreover, the interviewers’ different pre-understandings of primary healthcare work, may be seen as both an advantage and disadvantage in the communication with informants. Data was collected in 2014, but the results are likely to still be highly relevant since RNs’ telephone nursing work in Swedish primary healthcare has changed minimally over the past six years. The present study can henceforth serve as a valuable baseline measure to be compared with in future studies.

## Conclusion

Two overarching themes were found, namely professional challenges and professional support to answer the aim of describing RNs’ views of telephone nursing work with callers contacting PHCC regarding respiratory tract infection. The first theme contained three categories: Differentiate harmless from severe problems; Communicate for optimal patient information; Cope with caller expectations. The second theme included two categories: Use working tools and Use team collaboration. The present study is one of few international studies on telephone nursing at PHCCs. The results can be seen as a baseline to be compared with in future studies, regarding PHCC telephone nursing. The study deepens the understanding of telephone nursing as an important factor for decreasing RTI consultations with a GP, thus contributing to decreased antibiotic usage in Sweden. To cope with the challenges of telephone nursing in PHCCs, it seems important that managers in these organisations promote a systematic introduction for the use of DST and set aside time for inter- and intraprofessional discussions and feedback. The collegial support and team collaboration asked for is likely to get synergy effects such as better work environment and job satisfaction for both RNs and GPs. Future studies are needed to explore telephone nursing in PHCC in a broader sense to better understand the function and the effects in the complexity of primary healthcare.

## Abbreviations

CME: Continuous medical education
DST: Decision support tool
GP: General practitioner
PHCC: Primary healthcare centres
RN: Registered nurse
RTI: Respiratory tract infections
